# Incidence of Post-laparotomy Acute Kidney Injury Among Abdominal Trauma Patients and Its Associated Risk Factors at King Abdulaziz Medical City, Riyadh

**DOI:** 10.7759/cureus.44245

**Published:** 2023-08-28

**Authors:** Fahad K Alrzouq, Fares Dendini, Yousef Alsuwailem, Bader A Aljaafri, Abdulaziz S Alsuhibani, Ibrahim Al Babtain

**Affiliations:** 1 College of Medicine, King Saud Bin Abdulaziz University for Health Sciences, Riyadh, SAU; 2 Department of Research Office, King Abdullah International Medical Research Center, Riyadh, SAU; 3 Collage of Medicine, King Saud Bin Abdulaziz University for Health Sciences, Riyadh, SAU; 4 College of Medicine, King Saud Bin Abdulaziz University for Health Sciences College of Medicine, Riyadh, SAU; 5 Department of General Surgery, King Abdulaziz Medical City Riyadh, Riyadh, SAU

**Keywords:** risk factors, incidence, emergency laparotomy, abdominal trauma, acute kidney injury, post-laparotomy

## Abstract

Background

This research study investigates the prevalence of acute kidney injury (AKI) in trauma patients undergoing emergency laparotomies. AKI is a common complication in major surgeries and is associated with various adverse effects. The study aims to explore the relationship between AKI and other comorbidities in this specific context.

Methodology

This is a retrospective cohort study. All patients who had laparotomy after abdominal trauma at King Abdulaziz Medical City (KAMC) and met the inclusion criteria were included in the study. Nonprobability consecutive sampling was used. Data were collected by chart review using the Best-Care system at KAMC. Descriptive statistics were used to summarize and describe the characteristics of the study participants. Frequencies and percentages were calculated for categorical variables, such as comorbidities. For continuous variables, mean and standard deviations were calculated and tabulated. All statistical calculations were performed using IBM SPSS Statistics for Windows, Version 27.0 (IBM Corp., Armonk, NY, USA).

Results

This research study included 152 patients who underwent laparotomy, and the majority of patients (146, 96%) did not experience AKI. Several comorbidities were observed, with hypertension and diabetes being the most prevalent at 37 (24.3%) and 35 (23%), respectively. Intraoperative hypotension was experienced by 23 (15.1%) patients, while 129 (84.9%) did not have this issue. Norepinephrine was the most common vasopressor used (25.7%), followed by ephedrine and a combination of norepinephrine and epinephrine. Gender and age groups did not show significant associations with AKI, comorbidities like diabetes, heart failure, and chronic kidney disease (CKD) demonstrated significant relationships with AKI. There was no significant difference in eGFR and serum creatinine baseline levels between patients meeting AKI criteria and those who did not.

Conclusions

The low overall incidence of AKI in this patient population is encouraging. However, healthcare professionals must be aware of the significant impact of comorbidities such as diabetes, heart failure, and CKD on AKI development. Vigilant monitoring of postoperative kidney function, particularly serum creatinine levels within the first 48 hours, is essential for early detection and timely intervention. By understanding and addressing these risk factors, healthcare providers can take proactive steps to prevent and manage AKI in patients undergoing laparotomy, ultimately leading to improved patient outcomes and reduced healthcare costs.

## Introduction

Emergency laparotomies is an umbrella term that encompasses many emergent surgical procedures, whether it is trauma- or non-trauma-related. Although emergent laparotomies are lifesaving procedures, they are associated with a higher risk of mortality and morbidity compared to elective surgeries [1,2]. The preoperative, intraoperative, and postoperative statuses hold significant predictive value for the prognosis. In a study of postoperative complications following emergency laparotomy, it was found that age is one of the major risk factors affecting mortality. Specifically, 30% of patients aged 80 or older died within 28 postoperative days [3]. Additionally, the presence of other comorbidities such as hypertension, diabetes, coronary artery disease (CAD), and complications that occur during the surgery such as intraoperative hypotension and postoperative complications, including hemodynamic instability and nosocomial infections, greatly negatively influence the prognosis [4].

AKI is a clinical syndrome characterized by a precipitous decline in the function of the kidney and can affect its structure. It encompasses various etiologies, including kidney-specific diseases (e.g., acute interstitial nephritis, acute glomerular, and vasculitic renal diseases), nonspecific conditions (e.g., ischemia and toxic injury), and extrarenal pathology (e.g., acute postrenal obstructive nephropathy) [5]. According to the definition provided by Kidney Disease: Improving Global Outcomes (KDIGO), AKI is confirmed by any of the following: increase in serum creatinine (SCr) by 0.3 mg/dL (26.5 μmol/L) or more within 48 hours; increase in SCr to 1.5 times or more of the baseline, which is known or presumed to have occurred within the prior seven days; or urine volume less than 0.5 mL/kg per hour for six hours or more [5]. AKI is a common complication of patients undergoing major surgery and is associated with both short- and long-term complications and adverse effects such as ICU admission, longer hospital stay length, and chronic kidney disease (CKD) [3]. A study conducted in Portugal for noncardiac surgical patients (231 emergency surgery) showed that AKI accounts for 33% of postoperative intensive care unit (ITU) admissions [6]. Moreover, a UK cohort study done on 144 patients who underwent emergency laparotomy demonstrated that the incidence of renal complications was reported in 11.8% of the cases.

This study aims to identify the prevalence of acute kidney injury (AKI) in trauma patients undergoing laparotomy and the association between AKI in trauma-related emergency laparotomy and other comorbidities.

## Materials and methods

This study was conducted at King Abdulaziz Medical City (KAMC), a tertiary National Guard Hospital in Riyadh, Saudi Arabia. The hospital was instituted in May 1983, with a capacity of 3,133 beds approximately. It has a level I trauma center and serves as the trauma center in the region. The study is a retrospective cohort study that aims to investigate the Incidence of post-laparotomy AKI among abdominal trauma patients and associated risk factors at KAMC, Riyadh. It included all patients who had laparotomy after abdominal trauma. The sampling technique utilized for the study was nonprobability consecutive sampling, including all patients who met the inclusion/exclusion criteria. Data were collected by chart review using the Best-Care system at KAMC, and only the research team members collected the data. The collected data included patient demographics, comorbidities, quantitative factors such as estimated glomerular filtration rate (eGFR), serum creatinine baseline and after 48 hours from surgery, and surgery-related and after-surgery data such as the need for renal replacement therapy (RRT) and mortality.

The statistical analysis was conducted to examine the relationships and differences between variables in the study, as well as to determine the significance of the findings. The analysis involved both descriptive and inferential statistical tests.

Descriptive statistics were used to summarize and describe the characteristics of the study participants. Frequencies and percentages were calculated for categorical variables, such as comorbidities. For continuous variables, mean and standard deviations (SDs) were calculated and tabulated.

Additionally, the Fisher's Exact Test and Mann-Whitney U test were conducted to find the association of different variables with AKI. The nonparametric Mann-Whitney U test was chosen owing to the nonnormal distribution assessed by the application of the Kolmogorov-Smirnov test (*P *< 0.05). The significance level for all statistical tests was set at *P* < 0.05, indicating a 95% confidence interval. All statistical calculations were performed using IBM SPSS Statistics for Windows, Version 27.0 (IBM Corp., Armonk, NY, USA).

## Results

**Figure 1:**
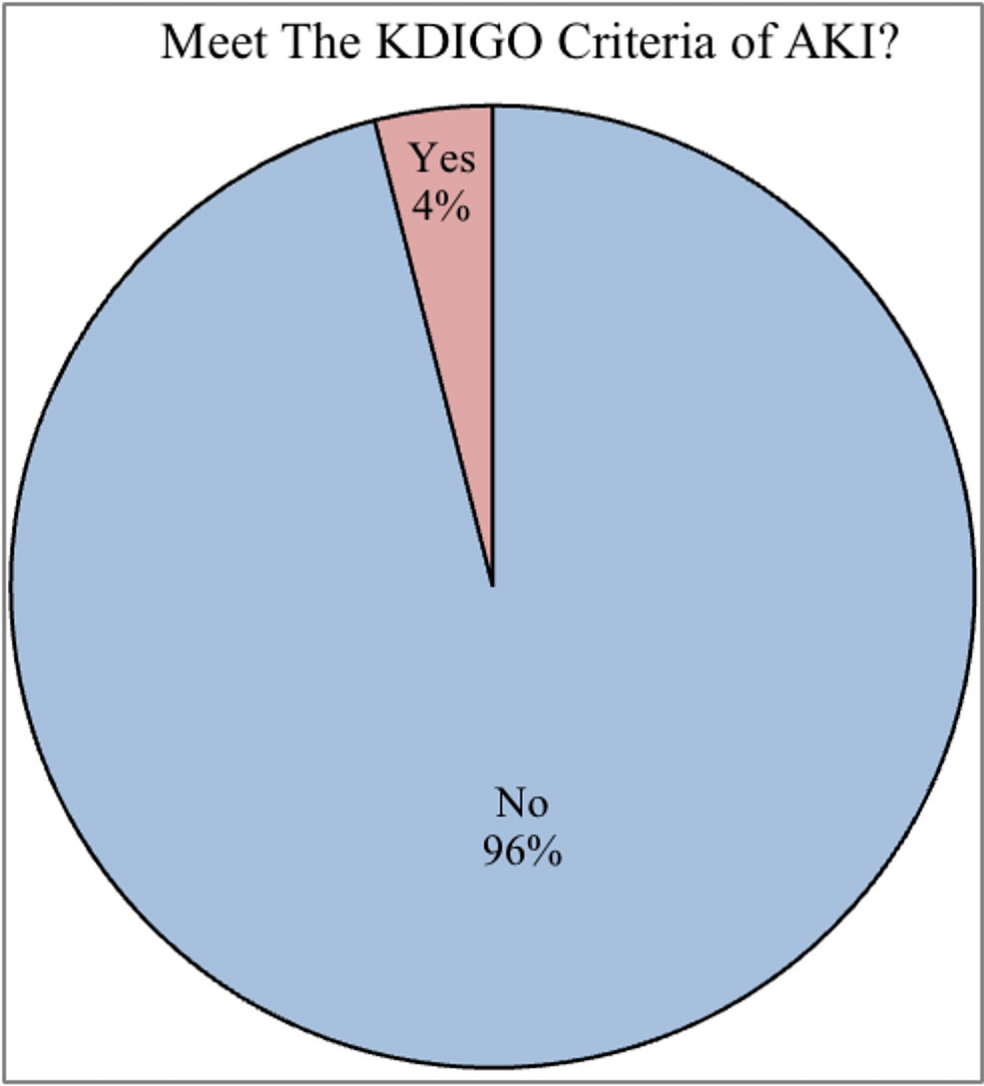
Patients meeting the KDIGO criteria of AKI. AKI, acute kidney injury; KDIGO, Kidney Disease: Improving Global Outcomes

A total of 152 patients were included in the analysis and evaluated for AKI. The majority of patients (146, 96%) did not meet the KDIGO criteria for AKI. On the other hand, a smaller proportion of patients, specifically 6 (4 %) individuals, met the criteria for AKI (Figure 1). 

Table 1 provides descriptive statistics on the demographic data and comorbidities related to AKI post-laparotomy. The study included a total of 152 patients. Among them, 56 (36.8%) individuals were female, while 96 (63.2%) individuals were male. Regarding age distribution, the majority of patients 79 (52%) were aged between 19 and 50 years, followed by 33 (21.7%) who were aged between 51 and 70 years, and the rest were aged between 0 and 18 and >70 years. Regarding body mass index (BMI) categories, the overweight and normal categories encompassed the majority of patients, accounting for 50 (32.9%) and 45 (29.6%), respectively. The comorbidities observed in the patients were hypertension in 37 (24.3%) individuals, diabetes in 35 (23%) individuals, and other comorbidities in a few patients.

**Table 1:** Descriptive statistics of demographic data and comorbidities related to AKI post-laparotomy. AKI, acute kidney injury; BMI, body mass index

Demographic data	Frequency (*n*)	Percentage (%)
Gender		
Female	56	36.8
Male	96	63.2
Age (years)		
0-18	20	13.2
19-50	79	52.0
51-70	33	21.7
>70	20	13.2
BMI		
Underweight	21	13.8
Normal	45	29.6
Overweight	50	32.9
Obesity Class 1	20	13.2
Obesity Class 2	8	5.3
Obesity Class 3	8	5.3
Comorbidities		
Hypertension	37	24.3
Diabetes	35	23
Heart failure	9	5.9
Previous stroke	4	2.6
Dyslipidemia	6	3.9

**Figure 2:**
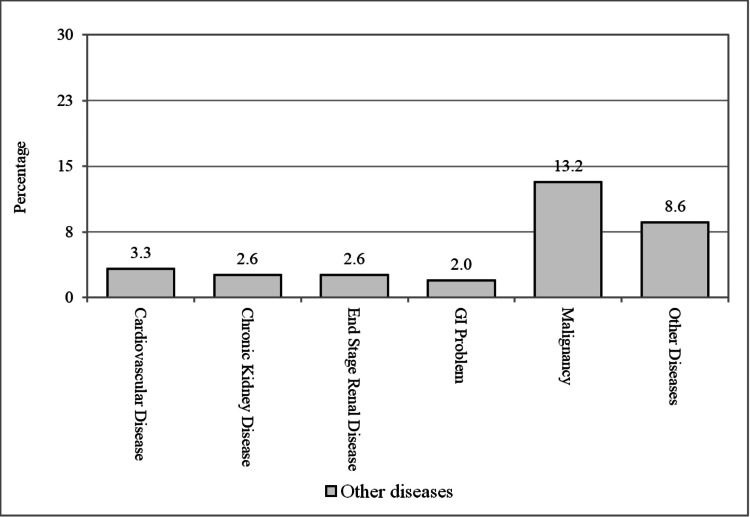
Patients having other diseases. GI, gastrointestinal

Regarding other comorbidities among the patients, 20 (13.2%) individuals had malignancy, 5 (3.3%) had cardiovascular disease, 4 (2.6%) had CKD, 4 (2.6%) had end-stage renal disease, and 4 (2.6%) had gastrointestinal (GI) problems. Additionally, 13 (8.6%) individuals had other unspecified diseases (Figure 2).

Table 2 provides descriptive statistics on surgical factors related to AKI post-laparotomy. Regarding the duration of surgery, the majority of patients fell into Class 1, with 68 (44.7%) individuals, followed by Class 2 with 65 (42.8%) individuals. A smaller proportion of patients were classified as Class 3 (10, 6.6%) and Class 4 (9, 5.9%). In terms of intraoperative hypotension, 23 (15.1%) individuals experienced hypotension, while the majority (129, 84.9%) did not. When considering vasopressor use, the most common vasopressor administered was norepinephrine, given to 39 (25.7%) individuals. Ephedrine was administered to 4 (2.6%) individuals, while a combination of norepinephrine and epinephrine was given to 4 (2.6%). In terms of blood loss, the majority of patients fell into Class 1, with 122 (80.3%) individuals, followed by Class 2 with 17 (11.2%) individuals. A smaller proportion of patients were classified as Class 3 (9, 5.9%) and Class 4 (4, 2.6%). Regarding post-laparotomy RRT, 9 (5.9%) individuals received this treatment, while the majority (143, 94.1%) did not. The mean eGFR was 99.6 ± 42.34 (range 13-218), the mean serum creatinine baseline was 89.7 ± 61.46 (range 34-475), and the mean serum creatinine after 48 hours from surgery was 76.7 ± 51.16 (range 31-343).

**Table 2:** Descriptive statistics of surgical factors related to AKI post-laparotomy. eGFR, estimated glomerular filtration rate; AKI, acute kidney injury; SD, standard deviation

Surgical factors	Frequency (*N* = 152), *n* (%)	Mean ± SD (Range)
Categorical surgical factors		
Duration of surgery (hours)		
Class 1 (up to 2)	68 (44.7)	
Class 2 (2-4)	65 (42.8)	
Class 3 (4-6)	10 (6.6)	
Class 4 (>6)	9 (5.9)	
Intraoperative hypotension		
Yes	23 (15.1)	
No	129 (84.9)	
Vasopressor use		
No	105 (69.1)	
Ephedrine	4 (2.6)	
Norepinephrine	39 (25.7)	
Norepinephrine and epinephrine	4 (2.6)	
Blood loss		
Class 1	122 (80.3)	
Class 2	17 (11.2)	
Class 3	9 (5.9)	
Class 4	4 (2.6)	
Post-laparotomy renal replacement therapy		
No	143 (94.1)	
Yes	9 (5.9)	
Numerical surgical factors		
eGFR		99.6 ± 42.34 (13-218)
Serum creatinine baseline		89.7 ± 61.46 (34-475)
Serum creatinine after 48 hours from surgery		76.7 ± 51.16 (31-343)

Table 3 provides information on qualitative factors associated with AKI post-laparotomy. The table presents the count and percentage of patients meeting or not meeting the KDIGO criteria for AKI based on various factors, along with the associated *P*-values. The analysis of gender showed that 2 (3.6%) females and 4 (4.2%) males met the KDIGO criteria for AKI, while the majority did not. The *P*-value (1.000) suggests no significant association of gender with AKI. Across different age groups, higher proportions of AKI were observed in the 51-70 years group (3, 9.1%) and the group aged more than 70 years (2, 10%), in comparison to the other age groups. However, the overall *P*-value (0.061) indicates a lack of statistical significance. Regarding comorbidities, diabetes, and heart failure showed a significant association with AKI (*P* < 0.05). For diabetes, 4 (11.4%) patients met the criteria, while 31 (88.6%) did not (*P *= 0.025). For heart failure, 2 (22.2%) patients met the criteria, while 7 (77.8%) did not (*P *= 0.041). CKD also showed a significant association with AKI. Two (50%) patients met the criteria, while 2 (50%) did not (*P *= 0.008). The rest of the comorbidities did not show any significant association. The remaining factors, including duration of surgery, intraoperative hypotension, vasopressor use, blood loss, post-laparotomy renal replacement therapy, and patient mortality, did not demonstrate significant associations with AKI.

**Table 3:** Qualitative factors associated with AKI post-laparotomy. ^*^P < 0.05, significant, Fisher's Exact Test. AKI, acute kidney injury; GI, gastrointestinal; BMI, body mass index

Qualitative factors		Meeting the KDIGO criteria of AKI?				*P*-value
		Yes		No		
		n	%	n	%	
Gender	Female	2	3.6	54	96.4	1.000
	Male	4	4.2	92	95.8	
Age (years)	0-18	0	0.0	20	100	0.061
	19-50	1	1.3	78	98.7	
	51-70	3	9.1	30	90.9	
	>70	2	10	18	90	
BMI	Underweight	0	0	21	100	0.354
	Normal	1	2.2	44	97.8	
	Overweight	4	8	46	92	
	Obesity Class 1	0	0	20	100	
	Obesity Class 2	0	0	8	100	
	Obesity Class 3	1	12.5	7	87.5	
Hypertension	No	4	3.5	111	96.5	0.634
	Yes	2	5.4	35	94.6	
Diabetes	No	2	1.7	115	98.3	0.025*
	Yes	4	11.4	31	88.6	
Heart failure	No	4	2.8	139	97.2	0.041*
	Yes	2	22.2	7	77.8	
Previous stroke	No	6	4.1	142	95.9	1.000
	Yes	0	0	4	100	
Dyslipidemia	No	6	4.1	140	95.9	1.000
	Yes	0	0	6	100	
Cardiovascular disease	No	5	3.4	142	96.6	0.185
	yes	1	20	4	80	
Chronic kidney disease	No	4	2.7	144	97.3	0.008*
	Yes	2	50	2	50	
Malignancy	No	5	3.8	127	96.2	0.578
	Yes	1	5	19	95	
GI problem	No	6	4.1	142	95.9	1.000
	Yes	0	0	4	100	
End-stage renal disease	No	6	4.1	142	95.9	1.000
	Yes	0	0	4	100	
Duration of surgery (hours)	Class 1 (up to 2)	2	2.9	66	97.1	0.458
	Class 2 (2 to 4)	3	4.6	62	95.4	
	Class 3 (4 to 6)	0	0	10	100	
	Class 4 (>6)	1	11.1	8	88.9	
Intraoperative hypotension	No	4	3.1	125	96.9	0.225
	Yes	2	8.7	21	91.3	
Blood loss	Class 1	5	4.1	117	95.9	0.739
	Class 2	1	5.9	16	94.1	
	Class 3	0	0	9	100	
	Class 4	0	0	4	100	
Vasopressor use	No	4	3.8	101	96.2	0.757
	Ephedrine	0	0	4	100	
	Norepinephrine	2	5.1	37	94.9	
	Norepinephrine and epinephrine	0	0	4	100	
Post-laparotomy renal replacement therapy	No	5	3.5	138	96.5	0.311
	Yes	1	11.1	8	88.9	
Patient died?	No	6	4.4	131	95.6	1.000
	Yes	0	0	15	100	

Table 4 presents quantitative factors associated with AKI post-laparotomy. The table includes the number of patients meeting the KDIGO criteria for AKI, the mean values, SD, and the associated *P*-values. There was no significant difference between patients meeting the AKI criteria and those who did not meet the criteria in eGFR (*P* = 0.165). For serum creatinine baseline, the mean/average level among patients meeting the AKI criteria was 97.33 ± 43.27, while among those who did not meet the criteria, it was 89.48 ± 62.19. The *P*-value of 0.345 indicates no significant difference between the two groups. However, the mean/average level of serum creatinine after 48 hours from surgery among patients meeting the AKI criteria was 190.00 ± 97.28, while among those who did not meet the criteria, it was 71.58 ± 41.98. The difference between the two groups was significant (*P* < 0.001).

**Table 4:** Quantitative factors associated with AKI post-laparotomy. ^*^P < 0.05, significant, Mann Whitney U test. AKI, acute kidney injury; eGFR, estimated glomerular filtration rate; SD, standard deviation

Meeting the KDIGO criteria of AKI?		*n* (%)	Mean ± SD	*P*-value
eGFR (Total 137)	Yes	6 (4.37)	75.33 ± 36.19	0.165
	No	131 (95.62)	100.72 ± 42.39	
Serum creatinine baseline (Total 150)	Yes	6 (4)	97.33 ± 43.27	0.345
	No	144 (96)	89.48 ± 62.19	
Serum creatinine after 48 hours from surgery (Total 138)	Yes	6 (4.34)	190.00 ± 97.28	<0.001^*^
	No	132 (95.65)	71.58 ± 41.98	

## Discussion

In this study, we investigated the incidence of AKI following laparotomy, exploring various demographic, comorbidity, surgical, and qualitative factors that may influence AKI development.

Our study examined 152 patients who underwent laparotomy, with 6 (4%) patients meeting the KDIGO criteria for AKI after laparotomy. This indicates a relatively low incidence of AKI in the immediate postoperative period in this particular patient population. The majority of patients (146, 96%) did not develop AKI following the surgical procedure.

Regarding the demographic data, we observed a higher proportion of males 96 (63.2%) compared to females 56 (36.8%) in the study cohort. Age distribution showed a significant number of patients 79 (52%) falling into the group of 19-50 years, and the most commonly encountered comorbidities were hypertension 37 (24.3%) and diabetes 35 (23%). The associations between gender and age groups with AKI did not reach statistical significance, suggesting that AKI development was not influenced significantly by these demographic factors.

However, comorbidities such as diabetes, heart failure, and CKD were found to be significantly associated with AKI. Patients with diabetes exhibited an 11.4% incidence of AKI, while those without diabetes had a much lower rate (*P *= 0.025). Similarly, heart failure showed a 22.2% incidence of AKI compared to 7.8% in patients without heart failure (*P *= 0.041). CKD also demonstrated a strong association with AKI (*P *= 0.008). These findings emphasize the importance of recognizing preexisting comorbidities, as they can play a critical role in the development of AKI following laparotomy [7].

Regarding the descriptive statistics on surgical factors, the duration of surgery was found to be statistically nonsignificant, with the majority of patients falling into Class 1 68 (44.7%) and Class 2 65 (42.8%). However, this finding is not consistent with previous studies that reported a positive correlation between prolonged surgery duration and AKI incidence. These studies showed that longer surgical durations might increase the risk of AKI due to prolonged exposure to anesthesia and potential hypoperfusion of the kidneys [8,9].

Intraoperative hypotension is another critical factor that may contribute to the development of AKI. In this study, 23 (15.1%) of patients experienced intraoperative hypotension, while the majority did not. Hypotension can lead to reduced renal blood flow, resulting in renal ischemia and subsequent injury [10]. The incidence of AKI in patients who experienced intraoperative hypotension should be compared to those who did not to determine if there is a significant association between AKI and intraoperative hypotension.

Vasopressor use, particularly norepinephrine, was administered to a significant proportion of patients 39 (25.7%). Previous studies have suggested that vasopressor use during surgery can affect renal perfusion and function, potentially contributing to AKI [11,12]. The impact of different vasopressors on AKI incidence should be explored further in future studies.

Blood loss during surgery is another factor that may influence the development of AKI. In this study, the majority of patients (80.3%) were categorized as Class 1 for blood loss. Significant blood loss can lead to hypotension and decreased renal perfusion, contributing to AKI development [13]. Comparing AKI incidence among patients with varying levels of blood loss may help confirm this association.

In this study, post-laparotomy RRT was required in 9 (5.9%) of the patients. The need for RRT indicates severe AKI and a higher risk of adverse outcomes [14]. Identifying factors associated with RRT requirements can help in risk stratification and timely intervention.

Regarding the qualitative factors associated with AKI post-laparotomy. Gender did not show a significant association with AKI, but contradicting other studies showed a significant association in both males and females depending on age groups as per this reference study [15]. The role of gender in AKI development remains controversial and warrants further investigation.

In terms of age, both the 51-70 years group and the more than 70 years group exhibited higher proportions of AKI. However, the overall *P*-value did not achieve statistical significance. Advanced age has been recognized as a risk factor for AKI in various studies [16,17], and a larger sample size might reveal significant associations in this study.

Comorbidities such as diabetes, heart failure, and CKD showed significant associations with AKI. Diabetic patients had a higher AKI incidence (11.4%), consistent with previous research that reviewed a case-matched retrospective study that showed the incidence of postoperative AKI in diabetes mellitus (DM) was 30.7%. Additionally, another study reviewed in the same research showed high DM prevalence in AKI with 19.6% [18]. Heart failure and CKD were also significantly associated with AKI, indicating the importance of recognizing these conditions as risk factors during preoperative evaluation [19].

Regarding the quantitative factors associated with AKI. There was no significant difference in eGFR between patients meeting AKI criteria and those who did not. This suggests that the baseline kidney function alone may not be sufficient to predict AKI occurrence, and other factors may be more influential [20].

However, the mean serum creatinine level after 48 hours from surgery was significantly higher in patients meeting the AKI criteria compared to those who did not. This indicates that the postoperative serum creatinine level can be a valuable indicator of AKI development and supports the findings of previous studies [21].

It is essential to acknowledge some limitations of our study. First, the relatively small sample size may have affected the statistical power to detect certain associations, particularly when examining rare events like AKI. Expanding the sample size in future studies could strengthen the analysis. Additionally, there might be some missing quantitative data that could influence the assessment of AKI occurrences. By placing more emphasis on gathering quantitative data in the future, we can potentially achieve a more accurate evaluation of AKI. Additionally, this study’s single-center design might limit the generalizability of the findings to other populations and healthcare settings.

## Conclusions

The low overall incidence of AKI in this patient population is encouraging. However, healthcare professionals must be aware of the significant impact of comorbidities such as diabetes, heart failure, and CKD on AKI development. Vigilant monitoring of postoperative kidney function, particularly serum creatinine levels within the first 48 hours, is essential for early detection and timely intervention. By understanding and addressing these risk factors, healthcare providers can take proactive steps to prevent and manage AKI in patients undergoing laparotomy, ultimately leading to improved patient outcomes and reduced healthcare costs.
